# Suitability of a Novel Diet for a Parasitic Wasp, *Cotesia plutellae*

**DOI:** 10.1673/031.013.8601

**Published:** 2013-09-17

**Authors:** Olalekan J. Soyelu

**Affiliations:** 1 Department of Zoology and Entomology, University of Fort Hare, Alice, South Africa; 2 Present address: Insect Physiology Laboratory, Department of Crop Production and Protection, Obafemi Awolowo University, Ile-Ife 220005, Osun State, Nigeria

**Keywords:** beebread, development time, feeding response, longevity, parasitism

## Abstract

The braconid *Cotesia plutellae* (Kurdjumov) (Hymenoptera: Braconidae) is a major solitary, larval endoparasitoid of the diamondback moth, *Plutella xylostella* (L.) (Lepidoptera: Plutellidae). The impact of dietary protein was investigated in the laboratory by comparing performance of *C. plutellae* on honey, which is commonly used to rear the parasitoid, to that on a novel diet made of honey and protein-rich beebread. *Cotesia plutellae* was highly stimulated by honey and honey-beebread, with a feeding response exceeding 95%, a level that is comparable with its responses to fructose, glucose, and sucrose. The ability of honey-beebread to support host-parasitoid colonies was also comparable with that of honey. However, parasitoids raised on honey-beebread suppressed diamondback moths in rearing cages 3 weeks before the honey-fed wasps. The development time of *C. plutellae* reared on honey with or without beebread showed no significant difference, but adult wasps lived longer on honey-beebread. Mean developmental periods from oviposition to pupation and from pupation to adult emergence were 8 and 6 days, respectively. Adult wasps raised on honey-beebread outlived their conspecifics that were raised on honey by at least 4 days. Honey-beebread showed potential as a good food for rearing *C. plutellae* in the laboratory, and its benefit in parasitoid production is discussed.

## Introduction

The mass production of insects for biological control of pests has become a common technique for permanent establishment, periodic colonization, or inundative releases of agents (Etzel and Legner 1999). The importance of natural enemies, particularly parasitoids, as biological control agents has fostered dietetic studies for the development of artificial diets as a way to augment numbers and reduce production costs in mass rearing facilities (Li 1992; Cônsoli and Parra 1997, 1999). Artificial diets for insects have been developed to maximize insect growth and reproduction by meeting or surpassing their minimum nutritional requirements. There is a wide range of diets and published formulations. Insects thrive on ideal artificial diets, making it possible to economically raise hundreds of thousands of insects to release in fields for management of damaging crop pests.

Honey is the most common diet used in the laboratory for rearing *Cotesia plutellae* (Kurdjumov) (Hymenoptera: Braconidae), but being mostly sugars (95–99% per dry matter; FAO 1996) with trace amounts of vitamins or minerals, it is assumed to be inadequate for maximum performance in a synovigenic parasitoid. The present study aimed at improving the quality of honey without hampering the performance of *C. plutellae* by adding a protein source, namely beebread, to honey to form a paste (honey-beebread), which was then used to rear *C. plutellae* in the laboratory. Beebread is a stable yellowishbrown mixture of pollen and nectar collected by the honeybee, *Apis mellifera*, and stored in honeycombs. It is formed when collected pollen undergoes lactic acid fermentation, a process that improves nutritional value of pollen and renders the end product more digestible (Loper et al. 1980). Unlike honey, beebread is rich in essential amino acids, carbohydrates, fatty acids, vitamins, minerals, trace elements, enzymes, and hormone precursors (FAO 1996).

*Cotesia plutellae* is the most common larval endoparasitoid attacking diamondback moth, *Plutella xylostella* (L.) (Lepidoptera: Plutellidae), in South Africa (Mosiane et al. 2003). It accounts for more than 80% of total parasitism on *P. xylostella* (Smith and Villet 2003), which is a destructive pest of cruciferous vegetables in many parts of the world. The parasitoid is synovigenic in nature (Soyelu 2010) and it always egresses from a stillliving L4 (the final instar) of *P. xylostella* to pupate externally, irrespective of the host instar at oviposition (Shi et al. 2002). Synovigenic females emerge with a fraction of their total egg complement and they continue to mature eggs for some time or throughout their adult life (Jervis et al. 2001). Many synovigenic parasitoids feed on host body fluids in order to achieve maximal lifetime reproduction, a phenomenon termed ‘host-feeding’ (Heimpel and Collier 1996). By feeding upon hosts, females can gain nutrients such as proteins and vitamins, acquiring resources for producing eggs (Collier 1995). However, this feeding habit is destructive, as parasitized hosts, often in considerable proportion, are usually not suitable for offspring development. Host-feeding can also cause significant mortality in host populations, in some cases causing more host deaths than by parasitism (Kidd and Jervis 1989). Provision of a protein supplement to an artificial diet could, therefore, be important in the diet of *C. plutellae* to reduce the need for host body fluid as a protein source.

The goal of this study was to provide a dietary protein that is available to *C. plutellae* without having adverse effects on *C. plutellae* feeding response, sustainability, reproduction, development, or longevity. It was hypothesized that an ideal protein additive to honey would enhance performance of *C. plutellae*. The study was divided into 3 parts. The first part assessed acceptability of the honey-beebread paste by comparing feeding response of *C. plutellae* to honey, honey-beebread, dilute sugar solutions, and sugardeprived sources. The second part compared sustainability of *P. xylostella-C. plutellae* colonies on honey and honey-beebread. The third phase compared rate of development and longevity of *C. plutellae* on honey and honey-beebread.

## Materials and Methods

### Food and chemical reagents

Honey produced by *A. mellifera* from Karoo wildflowers was purchased from Speelmanskop Honey, Cradock, South Africa. Beebread was obtained from honeycombs donated by Makana Meadery (www.iqhilika.co.za). One part of the cakelike beebread was crushed in 3 parts of honey (w/w), resulting in the honey-beebread. The insects were able to utilize beebread in this state. The sources and purity of sugars used are presented in Table 1. Yeager's physiological salt had the following composition (g/L dH_2_O): NaC1 10.93, KCl 1.57, CaCl_2_ 0.85, MgCl_2_ 0.17, and NaHCO_3_ 0.17.

### Experiment I: Feeding response

The method of Winkler et al. (2005) was used to test the feeding response of 2-day-old mated, unfed but water-satiated female *C. plutellae* to honey, honey-beebread, 1 M sugar solutions (fructose, glucose, sucrose, maltose, trehalose), physiological salt, and distilled water. To ensure water satiation at the time of the experiment, wasps were provided with soaked cotton wool for a period of 30 min prior to the trials. Subsequently, each wasp was transferred to a separate, inverted, 6.0 cm × 2.5 cm (height × diameter) glass vial with a tiny drop of test food in it. As soon as the wasp's tendency to walk upwards had brought it in contact with the test material, its feeding response was recorded. The reaction was scored as acceptance (if feeding lasted more than 5 sec) or rejection (feeding for less than 5 sec). Observations were made for a maximum of 10 min for each wasp, beginning at the time the parasitoid first came in contact with the food. Durations of successful feeding bouts within the period were summed-up to assess relative acceptance time to each treatment. Each treatment was assessed using 30–41 female wasps, and each individual was tested once. Feeding outcome was expressed as percent positive response to each treatment. Replicated feeding duration was then subjected to oneway analysis of variance (Program GLM, SAS Institute 1999), and sample means were compared among treatments using Duncan's multiple range tests.

### Experiment II: Host-parasitoid population dynamics in the laboratory

Larvae of *P. xylostella* and parasitoid cocoons were collected from cabbage fields in the vicinity of Alice (32° 46′ S, 26° 50′ E, 540 m a.s.l.), East Cape Province of South Africa, and emerging insects were used to establish standardized host-parasitoid colonies in December 2008. Colonies were established inside ten rearing cages (53 × ;53 × 53 cm); 5 cages were provided with honey while the other 5 were supplied honey-beebread. Each cage was started with 40 second instar *P. xylostella* larvae, 6 newly emerged *C. plutellae* (2 females, 4 males), and 24 cabbage seedlings (4 × 6 plastic cups). The parasitoids were removed from cages after 4 days, and each cage was supplied with 10 second instar larvae 13, 14, 19, and 20 days after cage establishment to serve as oviposition substrates for emerging parasitoids. Insect colonies were maintained in the laboratory at 25.1 ± 0.7° C (mean ± SD), 49.3 ± 8.3% RH, and with a 15:9 L:D photocycle. Food was streaked thinly on the interior top surface of the cages, while water was provided through a wick of cotton wool in water-filled glass vials. Cabbage seedlings grown in a compost medium were regularly supplied in the rearing cages to serve as host plant food for larvae. Control cages were established for each treatment, where *P. xylostella* was reared without the parasitoid.

Weekly performance assessment of *C. plutellae* on each treatment commenced 4 weeks after cage establishment. The number of *P. xylostella* and *C. plutellae* present in each cage was counted every Monday between 10:00 and 11:00 from 12 January to 28 September 2009. Correlation analysis was carried out to determine relationships between the number of moths and *C. plutellae* on each diet (SAS Institute 1999). Student's *t*-test was used to test the null hypothesis that equal number of moths/wasps emerged in honey and honey-beebread cages (GenStat for Windows 2007).

### Experiment III: Development and longevity

A sample of newly emerged parasitoids (< 24 hr) from parent insects that were fed honey or honey-beebread was used to set up 30 pairs (replicates) for each corresponding food treatment. Each pair (1 male and 1 female ) was confined in a 24.5 cm × 14.5 cm glass jar containing 2 or 3 cabbage seedlings infested with a batch of thirty second instar *P. xylostella* larvae. The *P. xylostella* larvae served as oviposition substrate for female parasitoids, and food (similar to that fed to parent waps) was provided to serve as a source of energy. Wasps were removed after 24 hr, and glass jars were examined on a daily basis for the presence of parasitoid cocoons. Cocoons collected were kept inside separate Petri dishes until parasitoid eclosion. Emerged parasitoids were maintained in separate jars and given food, similar to that fed to their parents, until death to assess longevity. The rate of development from egg to pupa was measured as the number of days from oviposition to cocoon appearance. The number of days from cocoon appearance to adult eclosion was also recorded for each wasp.

Longevity was also determined among freshly emerged wasps that were provided with water alone, physiological salt, and those that were left without any provision. Parasitoid development was compared between honey and honey-beebread only. Obtained data were analyzed using one-way analysis of variance (Program GLM, SAS Institute 1999) to determine diet effect on longevity. Sample means were then compared using Duncan's multiple range tests. The null hypotheses that rate of parasitoid development on honey was equal to that on honey-beebread, and that males do not outlive females, were tested using *t*-test procedure (GenStat for Windows 2007).

## Results

### Experiment I: Feeding response

**Figure 1.**
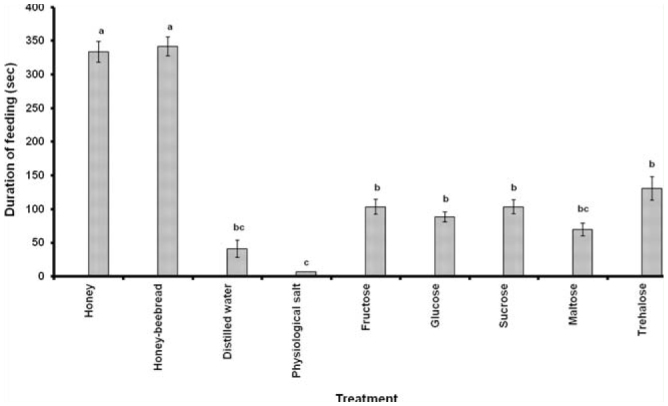
Duration of feeding (± SE) by water-satiated female *Cotesia plutellae* on honey, honey-beebread, specific sugars, physiological salt, and distilled water. Bars having the same letter are not significantly different at *p* ≤ 0.05. High quality figures are available online.

Honey, honey-beebread, sucrose, fructose, and glucose evoked > 90% feeding response, while maltose and trehalose elicited < 50% response in *C. plutellae*. The response to distilled water was < 10%, and physiological salt was the least stimulatory of the treatments, evoking an acceptance of < 5% (Table 2). In general, feeding occurred in discontinuous bouts, and the total time spent by each wasp within the 10 min of observation was summed up. The wasps fed for < 10 sec on physiological salt and < 50 sec on distilled water. Feeding duration on specific sugars (maximum 130 sec) was significantly lower than time spent (maximum 330 sec) on honey and honey-beebread (Figure 1). With the exception of maltose, all sugars tested led to longer durations of feeding than did the distilled water control.

### Experiment II: Host-parasitoid population dynamics in the laboratory

**Figure 2.**
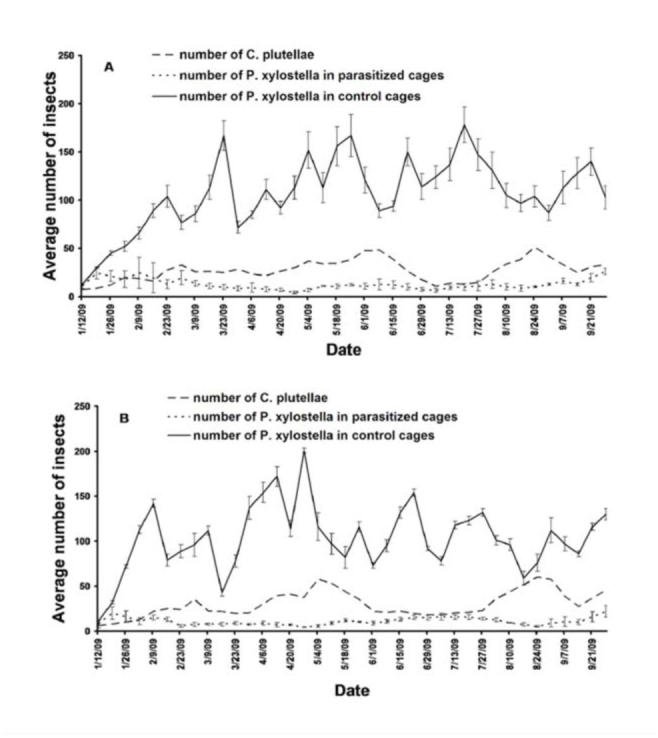
Relative abundance of *Cotesia plutellae* and *Plutella xylostella* in cages provided with (A) honey and (B) honey-beebread. *Plutella xylostella* population (± SE) was compared between test and control cages. High quality figures are available online.

The first set of *C. plutellae* emerged in established cages 13 days after larvae were parasitized. Emerging wasps suppressed the *P. xylostella* population, and this became evident in honey-beebread cages 3 weeks (February 2) before the same effect was observed in those fed honey alone (February 23) (Figure 2). The number of moths in control cages was consistently higher than the number in parasitized cages. The host-parasitoid population varied with diet over the rearing period, and peak population of *C. plutellae* was usually followed by a sharp decline, after which the number increased again to reach another peak. There was a significant negative correlation between the number of moths and wasps that emerged in honey-beebread cages (*c* = -0.38; df = 37; *p* = 0.019), while an insignificant relationship was observed in honey cages (*c* = 0.15; df = 37; *p* = 0.376). An equal number of *C. plutellae* emerged in honey and honey-beebread cages over the test period (*t* = 1.67; df = 37; *p* = 0.102), but the number of moths in honey cages was slightly higher than that in honey-beebread cages (*t* = 2.19; df = 37; *p* = 0.035).

### Experiment III: Development and longevity

Development times of *C. plutellae* eggs and larvae (from oviposition to pupation) and pupae (from pupation to adult emergence) were not significantly different on honey and honey-beeebread (eggs and larvae: *t* = 1.30; df = 539; *p* = 0.19; pupae: *t* = 1.50; df = 479; *p* = 0.14). Also, overall development times of *C. plutellae* from oviposition to adult emergence did not differ on the 2 diets (*t* = 1.67; df = 475; *p* = 0.17) (Table 3).

Adult male and female *C. plutellae* lived significantly longer on honey-beebread than they did on honey, whereas there was no significant difference between the lifespan of wasps that were not fed at all and those that were deprived of sugar sources (Table 4). Longevity of fed wasps was about ten-fold that of unfed conspecifics. Median longevity (day at which 50% of the initial number of wasps were still alive) was highest, and risk of starving to death was lowest, in wasps reared on honey-beebread. There was no sexual effect on longevity except among sugardeprived wasps, where females lived significantly longer than males.

## Discussion

A feeding response is elicited by stimulating gustatory chemoreceptors, which contain neurons that are specialized for either sugar, water, or salt (Nation 2008). In the present study, acceptance or rejection of a treatment was typically preceded by contact with mouthparts, suggesting a role of the mouth apparatus for sugar perception in parasitic Hymenoptera. However, involvement of taste receptors in other parts of the insect body cannot be ruled out. Honey and honey-beebread were highly stimulatory food sources, as were fructose, glucose, and sucrose. Maltose and trehalose could be categorized as moderately stimulatory sugars, while the physiological salt could be regarded as an antifeedant of *C. plutellae*. The poor acceptance of distilled water was due to the fact that wasps were water-satiated prior to the trials. The obtained results corroborate earlier ones, which showed that most hymenopterous parasitoids readily accept fructose, glucose, and sucrose, which are abundant in honey, nectar, and honey dew (Beach et al. 2003; Winkler et al. 2005). An excellent feeding response to component sugars is imperative to successful utilization of a food source. Positive feeding response is a vital behavior in insect rearing, and a good fit between response and longevity has been shown for a number of insects (Irvin et al. 2007).

Feeding durations on honey and honey-beebread were longer than on sugar solutions, suggesting a reduced rate of fluid intake due to high viscosity (Wyckhuys et al. 2008). An exponential increase in viscosity is a major characteristic of increasing sugar concentration (Borrell 2006), a factor that affects the handling of food in insects (Heyneman 1983). Though a parasitoid is more satisfied the longer it feeds on a food source, the prolonged stay may be disadvantageous in the field, as a wasp spending a long time on a highly concentrated sugar source could be an easy target for predators (Morse 1986). It might also have a strong impact on daily time allocation of the wasp, as less time is devoted to host searching and egg laying activities. Application of diluted supplementary food spray, therefore, becomes pertinent in this respect, as a high amount of sugar can be consumed in a relatively short feeding time when diluted solutions are applied in the field (Siekmann et al. 2001).

Honey-beebread sustained *P. xylostella-C. plutellae* colonies in the laboratory in a manner that is comparable with honey. However, wasps in honey-beebread cages attained adequate fitness faster than those in honey cages, and the numbers of diamondback moths were subdued much earlier in the former. This disparity could be due to quality of diet, as honey-beebread is more nutritious than honey. It is evident that pest population remained perpetually low in test cages, indicating that the parasitoid was successfully established in the 2 treatments. The negative correlation between the number of diamondback moths and *C. plutellae* in test cages together with a consistently higher number of *P. xylostella* in control cages confirmed that reduction in pest population was due to parasitism.

The sharp decline in the number of *C. plutellae* after each peak population and the subsequent increase could be attributed to the phenomenon of density-dependent parasitism. Mortality factors, such as parasitism, acting on an insect population can cause 3 possible dynamic changes. They can: (i) affect the average population density, (ii) induce fluctuations in numbers, and (iii) contribute to the regulation of population numbers (Kidd and Jervis 2007). For a factor to regulate, the strength of its action must be dependent on the density of the population affected. Thus, its proportional effect has to be greater at high population densities and smaller at low densities. Given that the parasitoid needs its host to multiply, it would be disadvantageous to the parasitoid to eradicate the host in its entirety. In the present study, *C. plutellae* attacked more larvae at high larval densities, and when the larval population was depleted, the parasitoid reduced the rate of parasitism. This reduction allowed diamondback moth larvae to increase in number, accompanied by an increase in rate of parasitism, until the peak attack was attained and the process of number regulation re-started. The emergence of wasps from eggs laid at high larval densities gave rise to the peaks (Figure 2), which were followed by the sharp descent recorded for fewer wasps that emerged from a reduced number of eggs laid at low larval densities. It could, therefore, be said that this regulatory activity of the parasitoid partly kept colonies alive in the laboratory.

The development time of *C. plutellae* did not vary with or without protein supplement in the diets. It took an average of 8 days from oviposition to pupation, and an additional 6 days before adults eclosed on both diets. The average development time of *C. plutellae* from oviposition to adult emergence was, therefore, about 14 days. The life span of an individual insect can be divided into 2 phases: (i) the development from hatching of the egg until adult eclosion, and (ii) the period of adult life, usually referred to as longevity (Blackburn 1991). The subject of longevity is crucial to the success of biological control because (a) the longer a male can live, the more females he can inseminate, and therefore the more eggs he can fertilize; and (b) the longer a female can live, the more eggs she will lay (Roitberg et al. 2001; Rivero and West 2002). The results of our study showed that sugarsatiated wasps lived significantly longer than their sugar-deprived cohorts. This observation is in agreement with Onagbola et al. (2007), who noted a significant reduction in longevity of unfed wasps. Starved wasps lived for just 2 days, suggesting that resources acquired during immature development were rapidly exhausted for somatic maintenance. On average, food availability increased parasitoid longevity by a factor of 10 relative to wasps that were not fed at all. However, *C. plutellae* lived longer on honey-beebread than it did on honey, while physiological salt appeared to be the most unsuitable food for this parasitoid. Since beebread is very rich in essential amino acids, it is reasonable to suggest that the presence of amino acids led to the longer life span recorded for the *C. plutellae* that were raised on honey-beebread. Rojas et al. (1995) observed an increase in longevity of *Bracon thurberiphagae* reared on *Heliothis virescens* when the adult diet (30% solution of 50:50 glucose:fructose) was fortified with several amino acids. Provision of protein sources assupplemental food could be the key to improving mass rearing efficiency of *C. plutellae*. In the field, where hosts are presumably more difficult to find and the rate of parasitoid oviposition may, therefore, be lower, it seems likely that longevity would have a stronger influence on parasitoid fitness.

General life history models predict that age at maturity and adult lifespan should be positively correlated (Stearns 1992), but studies have shown that hymenopteran parasitoids are an exception. In the present study, diet affected longevity, but it did not influence development time. Eijs and van Alphen (1999) and Seyahooei et al. (2011) observed no correlation between development time and adult lifespan among 5 species of the parasitoid genera *Leptopilina* and *Asobara*. Specifically, the latter reported that the shortest-lived species, *Asobara japonica*, had a development time that was longer than that of *A. pleuralis* which, on the contrary, had an adult lifespan that was about 4 times longer. Laboratory bioassays also showed a negative relationship between longevity and metabolic rate in *Asobara*. It was, therefore, concluded that lifespan and development time in parasitoids are genetically and physiologically uncoupled. This allows adult lifespan to evolve independently from development rate. It was suggested that metabolic rate and the ability to use adult food could determine adult lifespan but not development time.

In conclusion, these results showed that honey-beebread is a suitable supplementary food that could be used in place of pure honey for rearing *C. plutellae*. The excellent feeding response elicited by this diet is an indication that the addition of beebread to honey did not affect attraction to the food. The physiological salt caused a drastic reduction in feeding response of *C. plutellae*, indicating a need for caution when formulating artificial diets for this parasitoid. The suitability of honey-beebread for rearing *C. plutellae* in the laboratory has the potential to greatly expand the use of biological control agents in agriculture while reducing dependence on chemical products.

**Table 1.**
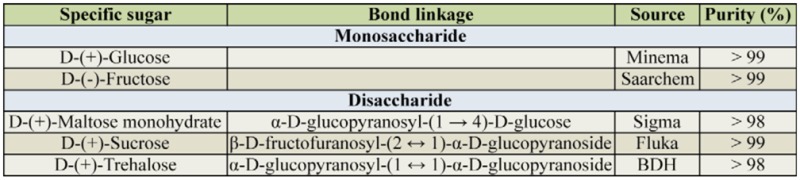
Sources and purity of sugars used in the feeding experiment.

**Table 2.**
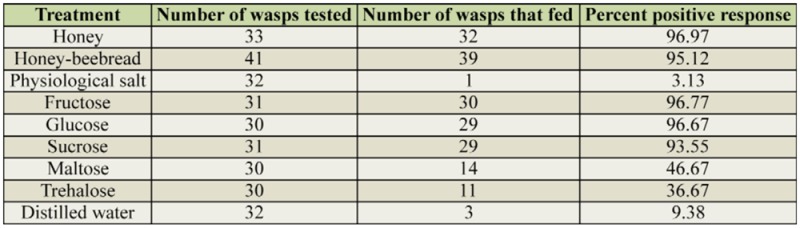
Feeding response of water-satiated *Cotesia plutellae* to honey, honey-beebread, physiological salt, and specific sugars compared to distilled water during 10 min observation periods.

**Table 3.**
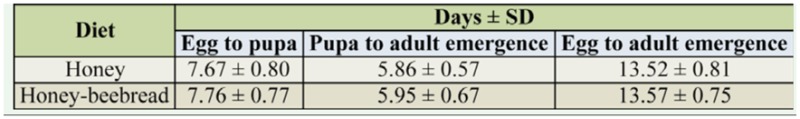
Development time of *Cotesia plutellae* reared on honey and honey-beebread.

**Table 4.**
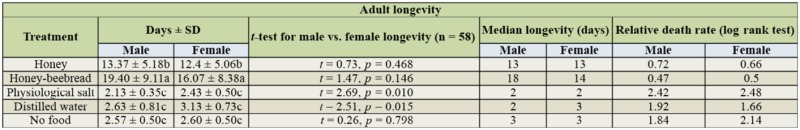
Indices of adult *Cotesia plutellae* longevity on 5 nutritional treatments. Values in the same column followed by the same letter are not significantly different at *p* ≤ 0.05.
